# Calprest ELISA vs. Liaison^®^ Chemiluminescence: Evaluating Accuracy, Efficiency, and Clinical Utility in Fecal Calprotectin Testing

**DOI:** 10.3390/biomedicines14010143

**Published:** 2026-01-10

**Authors:** Joško Osredkar, Nina Ekart, David Drobne

**Affiliations:** 1 Institute of Clinical Chemistry and Biochemistry, University Medical Centre Ljubljana, Zaloška cesta 2, 1000 Ljubljana, Slovenia; josko.osredkar@kclj.si (J.O.); nina.ekart@gmail.com (N.E.); 2 Faculty of Pharmacy, University of Ljubljana, Aškerčeva 7, 1000 Ljubljana, Slovenia; 3 Division of Internal Medicine, Department of Gastroenteorology, University Medical Centre Ljubljana, Zaloška cesta 2, 1000 Ljubljana, Slovenia; 4 Faculty of Medicine, University of Ljubljana, Vrazov trg 2, 1000 Ljubljana, Slovenia

**Keywords:** ulcerative colitis, fecal calprotectin, statistical analysis, endoscopy, ELISA, CLIA, endoscopic Mayo index

## Abstract

**Background**: Ulcerative colitis (UC) management relies on accurately assessing disease activity. Fecal calprotectin (FC) is a promising non-invasive biomarker, but method-specific differences in measurement can affect interpretation. **Objective**: To compare the performance of Calprest ELISA and DiaSorin Liaison CLIA in measuring FC concentrations and their correlation with endoscopic findings in UC. **Methods**: Stool samples from 40 UC patients were analyzed using both methods, with 138 samples collected across three clinical timepoints. Spearman’s correlation, Wilcoxon test, Bland–Altman analysis, and ROC curves were used to evaluate method agreement and diagnostic performance relative to Mayo endoscopic scores. **Results**: A total of 135 paired results showed strong correlation (ρ = 0.795, *p* < 0.001) but significant inter-method differences (*p* = 0.039). Liaison tended to yield higher FC values. ROC analysis established optimal cut-offs for detecting endoscopic remission and active disease: 47.95/69.55 µg/g (Liaison) and 65/125 µg/g (Calprest). Calprest demonstrated slightly better diagnostic accuracy. **Conclusions**: Both methods are reliable for monitoring UC activity. Calprest offers greater dynamic range, while Liaison excels in automation and speed. Method-specific thresholds should guide clinical interpretation to ensure accurate disease monitoring.

## 1. Introduction

Ulcerative colitis (UC) is a chronic inflammatory bowel disease (IBD) characterized by mucosal inflammation confined to the colon [1]. Clinical management relies heavily on assessing disease activity to guide treatment and monitor therapeutic response. While colonoscopy remains the gold standard for evaluation, it is invasive, costly, and not well tolerated by patients. Non-invasive biomarkers such as fecal calprotectin (FC) have emerged as reliable surrogates for mucosal inflammation, correlating well with endoscopic findings [2].

FC is a calcium- and zinc-binding protein abundantly present in neutrophils. During intestinal inflammation, activated neutrophils infiltrate the mucosa and release FC into the lumen. Its measurement in stool has proven clinically useful for differentiating IBD from functional disorders like irritable bowel syndrome (IBS), and for assessing disease activity in UC [2,3]. Several commercial assays exist for FC quantification, including enzyme-linked immunosorbent assays (ELISA) and chemiluminescent immunoassays (CLIA), each with distinct operational and analytical characteristics [4,5,6,7]. The characteristics of the methods compared in our study are shown in Table 1.

This study compares two widely used methods for FC determination: the Calprest ELISA and the DiaSorin Liaison CLIA. Our objective was to evaluate their analytical agreement and clinical correlation with endoscopic disease activity in patients with UC, as assessed by the Mayo endoscopic subscore. By comparing these platforms, we aim to provide guidance for laboratories and clinicians regarding method selection for routine monitoring of UC.

## 2. Materials and Methods

We included 40 patients (24 women, 16 men) with established UC, aged 20 to 80 years. All patients included in the study were receiving biologic therapy. This inclusion criterion was selected to evaluate assay performance specifically within the context of routine therapeutic monitoring, where accurate detection of residual inflammation is critical for treatment optimization. A total of 138 stool samples were collected at three treatment timepoints: pre-biologic therapy, 6–10 weeks after initiation, and at follow-up (up to 50 weeks). The follow-up samples primarily represent patients in the maintenance phase of biologic therapy, capturing a real-world spectrum of therapeutic response. Endoscopic scores were obtained within 40 days of sample collection. Data of included patients and clinical characteristics are presented in Table 1.

### 2.1. Calprotectin Measurement

Patients were instructed to collect stool at home and provide two aliquots in sterile containers. Samples were delivered to the laboratory as soon as possible and stored at −80 °C until analysis to preserve biomarker stability. Before testing, frozen samples were thawed at room temperature, homogenized, and processed according to the manufacturer’s instructions for each assay. Fecal calprotectin concentrations were determined using both the Calprest ELISA (Eurospital, Trieste, Italy) and the DiaSorin Liaison CLIA (Saluggia, Italy) platforms, strictly following the validated protocols provided by the manufacturers.

Calprest ELISA is a manual assay employing polyclonal antibodies and colorimetric detection. Liaison CLIA uses chemiluminescent detection with automated extraction and quantification. For both methods, the upper analytical limit was capped at 800 µg/g to normalize comparisons.

Study was approved by the National Medical Ethics Committee (approval number: 0120-232/2022). All participants provided written informed consent. The study was conducted in accordance with the Declaration of Helsinki

### 2.2. Statistical Analysis

Data distribution was first evaluated using the Shapiro–Wilk test. Continuous variables are presented as mean ± standard deviation (SD) for normally distributed data, or as median with interquartile range (IQR) when non-normally distributed. Categorical variables are reported as frequencies and percentages.

Agreement between Calprest ELISA and Liaison CLIA results was assessed using Spearman’s rank correlation coefficient (ρ) to evaluate monotonic associations and Bland–Altman plots to visualize inter-method bias and limits of agreement. Paired comparisons of FC concentrations were performed with the Wilcoxon signed-rank test.

For clinical validation, FC results were stratified according to Mayo endoscopic subscores. Associations between categorized FC values and disease activity were analyzed using the chi-square test. Diagnostic performance was evaluated using receiver operating characteristic (ROC) curves, with the area under the curve (AUC) calculated for remission versus active disease, and for mild versus moderate-to-severe inflammation. Optimal cut-off values were determined using the Youden Index (sensitivity + specificity − 1).

All statistical tests were two-tailed, and a *p*-value < 0.05 was considered statistically significant. Analyses were performed using MedCalc (Acacialaan 22, 8400 Ostend, Belgium, version 20.011-64-bit).

### 2.3. Endoscopic Assessment

Endoscopies were performed as full colonoscopies or flexible rectosigmoidoscoipes. Mucosal inflammation was assessed using endoscopic Mayo score [8].

## 3. Results

### 3.1. Comparison of Fecal Calprotectin Values

A total of 135 paired stool samples were analyzed using both Calprest ELISA and Liaison CLIA methods (Table 2). Descriptive statistics (Table 3) revealed higher median and mean FC values measured by Liaison (median 149 µg/g; mean 288 µg/g) compared to Calprest (median 102 µg/g; mean 242 µg/g). Despite this systematic difference, a strong positive correlation was observed between FC values measured by Liaison and Calprest (Spearman’s ρ = 0.795, *p* < 0.001), based on 135 paired samples. This indicates that the two methods provide consistent rank-ordering, despite differences in absolute values.

A Wilcoxon signed-rank test confirmed a statistically significant difference in paired FC values (*p* = 0.039), suggesting that the methods are not interchangeable at an absolute level, particularly at higher concentrations.

### 3.2. Method Agreement Analysis

**Figure 1:**
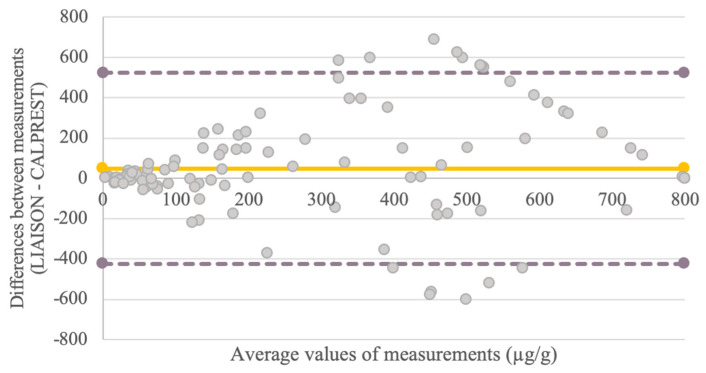
Bland–Altman Plot Comparing FC Concentrations Measured by Liaison and Calprest Methods. The plot shows the mean difference between the two methods with limits of agreement. Liaison values were systematically higher, particularly at elevated FC concentrations.

The Bland–Altman plot (Figure 1) revealed a mean positive bias of 49.6 µg/g in Liaison measurements relative to Calprest. Agreement between the methods was stronger at lower FC concentrations (<300 µg/g), while variability increased with higher values, though these still consistently reflected active inflammation.

### 3.3. Correlation with Endoscopic Mayo Scores

**Figure 2:**
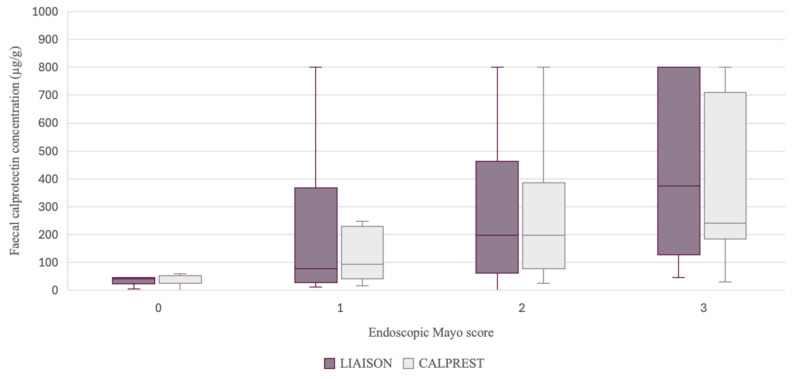
Median Fecal Calprotectin Concentrations by Mayo Endoscopic Score for Calprest and Liaison. Boxes represent first and third quartile. Lower and upper whiskers represent minimum and maximum. Median FC values for each Mayo category (0–3) illustrate a consistent rise in FC concentration with increasing inflammation severity. Liaison showed wider variability at higher scores.

Fecal calprotectin levels were compared against endoscopic Mayo subscores to evaluate clinical relevance. Median FC values increased progressively with higher Mayo scores for both methods (Figure 2). The strongest agreement was observed in Mayo 0 and 1 categories, where Calprest demonstrated narrower interquartile ranges, while Liaison exhibited greater variability, particularly in severe inflammation (Mayo 3).

Chi-square analysis confirmed a statistically significant association between FC concentration categories (<100 µg/g vs. ≥100 µg/g) and Mayo inflammation status for both Liaison (*p* = 0.014) and Calprest (*p* = 0.036).

### 3.4. Diagnostic Performance and Threshold Determination

**Figure 3:**
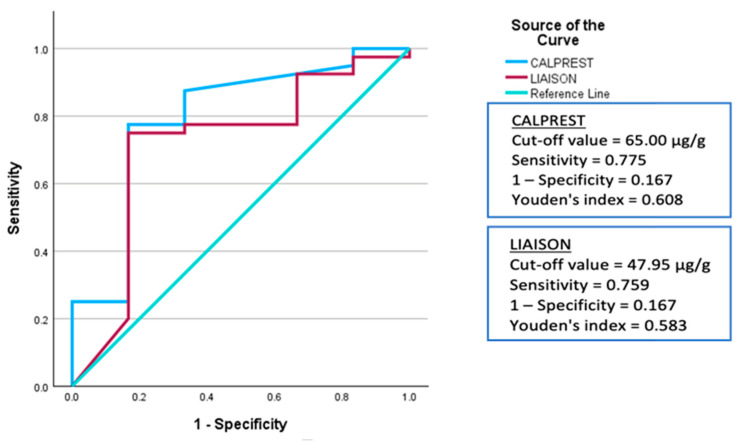
ROC Curves for Identifying Endoscopic Remission Using Calprest and Liaison Methods. ROC analysis of both assays in detecting endoscopic remission (Mayo 0). Calprest demonstrated higher overall diagnostic accuracy.

ROC analysis was performed to determine method-specific cut-off values for identifying endoscopic remission (Mayo 0) and active inflammation (Mayo 2–3). For remission, optimal thresholds were 47.95 µg/g for Liaison (Youden index = 0.583) and 65 µg/g for Calprest (Youden index = 0.608), as shown in Table 4. The Calprest method achieved slightly higher diagnostic accuracy (AUC = 0.794) compared to Liaison (AUC = 0.717) (Figure 3). Although Calprest demonstrated a numerically higher AUC compared to Liaison, a formal statistical comparison of the ROC curves (e.g., DeLong test) was not performed, as the primary objective was to define method-specific thresholds rather than establish the superiority of one diagnostic platform.

**Figure 4:**
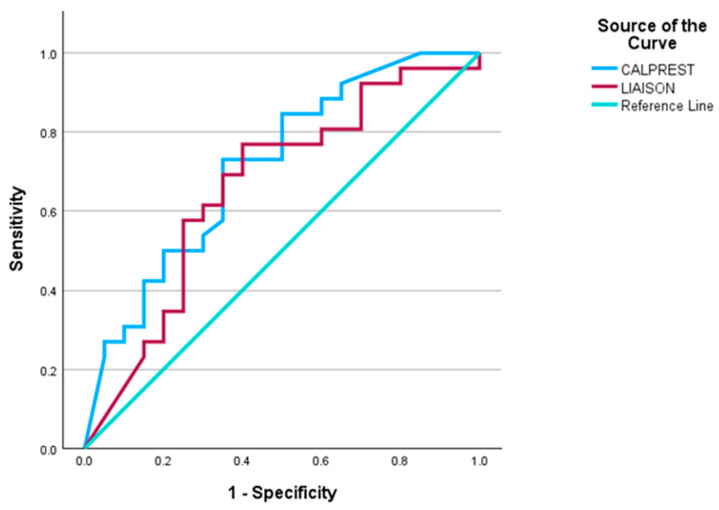
ROC Curves for Differentiating Endoscopic Improvement from Active Inflammation. ROC analysis for distinguishing Mayo 0–1 (remission/improvement) from Mayo 2–3 (active disease). Both methods performed less accurately in this comparison.

When distinguishing endoscopic improvement (Mayo 0–1) from active inflammation (Mayo 2–3), diagnostic performance decreased for both methods. Cut-off values of 69.55 µg/g (Liaison) and 125 µg/g (Calprest) yielded lower Youden indices and AUCs (Figure 4), indicating reduced discriminative ability in this context.

The Bland–Altman analysis (Figure 1) revealed a mean positive bias of 49.6 µg/g, with the Liaison method consistently producing higher FC values. Agreement was stronger at lower FC levels, while greater dispersion was observed above 300 µg/g, where both methods still reliably indicated inflammation.

Median FC levels increased across Mayo scores 0 to 3 for both assays (Figure 2). Calprest showed tighter interquartile ranges, while Liaison exhibited broader variability, particularly in Mayo 3, suggesting higher sensitivity to extremes of inflammation.

ROC curve analysis (Figure 3) showed that both methods effectively differentiated remission from active disease. Calprest yielded an AUC of 0.794 vs. 0.717 for Liaison, supporting slightly superior diagnostic performance.

When evaluating endoscopic improvement (Mayo 0–1 vs. 2–3), the diagnostic performance declined for both assays (Figure 4), with AUCs of 0.714 (Calprest) and 0.622 (Liaison), indicating a lower discriminative ability between mild and moderate inflammation.

## 4. Discussion

Our results demonstrate the efficacy of Liaison CLIA and Calprest ELISA in assessing fecal calprotectin (FC) and its correlation with endoscopic disease activity in ulcerative colitis (UC) patients. Although there was a close connection between the two assays, Liaison consistently produced higher FC concentrations, indicating that they were not interchangeable at the absolute value level. This inter-method bias is in line with earlier research that commercial FC assays differed in clinically significant ways even though their correlation coefficients were high [9].

Each method’s clinical value is further supported by its diagnostic accuracy in identifying active inflammation and endoscopic remission. Our remission cut-off values (47.95 µg/g for Liaison and 65 µg/g for Calprest) are similar to those of other studies, which depending on the method have often set the remission threshold between 50 and 100 µg/g [10,11]. For remission prediction, Calprest produced a marginally higher AUC than Liaison, which is in line with its wider dynamic range and prior application in IBD research settings.

Crucially, the Bland–Altman analysis reveals that the two tests exhibit the best agreement at lower FC concentrations, which are clinically essential for detecting remission. However, more variability surfaced, particularly with Liaison, at higher concentrations—relevant in active or refractory UC. This could affect the monitoring of patients with severe illness, as significant variations may affect the choice of treatment.

These discrepancies are probably caused by different methods. Calprest is a manual ELISA that is renowned for its accuracy in controlled laboratory settings and has a broader quantification range. Liaison, in comparison, has a little smaller analytical range but offers complete automation, which makes it appealing for high-throughput clinical applications. Recent assessments by Vicente-Steijn R et al. (2020) and Pelkmans LPJ et al. (2019) made similar findings, stressing the significance of assay selection in terms of laboratory workflow and result standardization [12,13].

These findings underscore a broader issue in calprotectin testing: the significant variability between commercial immunoassay kits. Due to the lack of an international reference standard, results from different manufacturers are not directly interchangeable. This confirms that clinical centers must validate method-specific cut-offs to avoid misinterpretation of disease activity when switching between assay platforms.

The clinical implication is obvious: when interpreting FC levels across platforms, labs and clinicians need to exercise caution. On one platform, a measurement of 70 µg/g might be seen as being within the remission range, but on another, it might be interpreted as indicating inflammation. Diagnostic consistency would be increased by implementing platform-specific thresholds or, preferably, standardized international standards. This is the direction of ongoing work by IBD biomarker working groups and ECCO [14].

Real-world clinical sample, paired technique comparisons, and connection with validated endoscopic rating are some of our study’s strong points. Its single-center approach and lack of histology proof of remission are drawbacks, too. Further research is necessary to address the unresolved issue of intra-individual diversity in FC, even within the same illness stage.

Future research should focus on developing cross-platform conversion equations or calibration factors and prospective multicenter validation of method-specific cut-offs. Furthermore, recent studies in digital health have demonstrated that incorporating FC into machine learning models for forecasting illness recurrence or biologic response could be a helpful approach [15].

In summary, although Calprest and Liaison are both useful instruments for evaluating UC activity, it is crucial to carefully evaluate their technical features and clinical setting. Enhancing the dependability of this crucial non-invasive biomarker still requires standardizing FC interpretation across systems.

Our study focused exclusively on fecal calprotectin due to its high specificity for intestinal inflammation compared to serum biomarkers. While serum calprotectin and other systemic markers such as CRP or leukocyte counts can provide complementary information, they lack the direct correlation with mucosal healing that fecal markers offer. Future studies integrating a multi-marker panel could further refine non-invasive monitoring algorithms.

### 4.1. Study Limitations

This study has several important limitations that should be considered when interpreting the results.

First, the relatively small sample size (n = 40 patients, 135 paired measurements) and single-center design may limit the generalizability of findings to other centers or populations with different UC demographics or disease phenotypes.

Second, a notable limitation of our study is the variable time interval between stool sampling and endoscopic evaluation, which extended up to several weeks in some cases. Ideally, samples should be collected within 7 days of endoscopy to minimize temporal discrepancies in disease activity. While this reflects real-world clinical logistics, we acknowledge that a tighter collection window would likely yield stronger correlations and higher diagnostic accuracy. It is known that FC can fluctuate over time, and temporal misalignment could weaken the observed correlations, particularly in patients with rapidly changing disease activity.

Third, the patient cohort consisted exclusively of individuals receiving biologic therapy (100% on biologics), which may not be representative of the general UC population or those receiving conventional therapeutic approaches. This may limit the applicability of our cut-off values to untreated or conventionally treated patients. Fourth, we did not perform histological assessment of mucosal inflammation, and therefore relied exclusively on endoscopic Mayo scoring, which, while validated and widely used, may not capture subclinical microscopic inflammation. Some patients with endoscopic remission may have residual histological inflammation, potentially affecting interpretation of the remission cut-offs.

Fifth, intra-individual FC variability, even within stable disease states, is well recognized in IBD literature but was not formally addressed in this analysis. FC values can be influenced by factors including concurrent infections, non-steroidal anti-inflammatory drug use, diet, and gut microbiota composition—variables not controlled for in this study. Finally, the normalization of measured values to 800 µg/g for both assays, while practical for comparison purposes, may have obscured true differences in the measurement of extremely high FC concentrations in severely inflamed colonic mucosa. These limitations suggest that larger, multicenter studies with more diverse patient populations and standardized protocols are needed to refine and validate the method-specific thresholds proposed here.

### 4.2. Clinical Implications

Despite these limitations, the findings have substantial practical relevance for clinical laboratories and gastroenterologists managing UC. The two assays differ meaningfully in their operational characteristics and should be selected according to specific laboratory needs and clinical context. Liaison CLIA offers significant advantages in workflow efficiency: fully automated sample preparation, rapid assay time (~35 min), continuous loading capacity, and high throughput suitable for large-volume clinical laboratories. These features make Liaison ideal for institutions requiring fast turnaround times and high sample volume processing.

Conversely, Calprest ELISA provides a broader analytical range (30–1800 µg/g), which is clinically advantageous for capturing the full spectrum of disease activity without sample dilution, and demonstrated marginally superior diagnostic accuracy for remission detection (AUC = 0.794). Calprest may therefore be preferable in research settings, specialized IBD centers, or laboratories where analytical range and precision at higher FC values are priorities.

Critically, this study highlights that FC values cannot be used interchangeably between platforms without risk of misclassification. A measurement of 70 µg/g on Liaison might be interpreted as remission using its cut-off (47.95 µg/g), whereas the same biological sample measured on Calprest might exceed its remission threshold (65 µg/g). To avoid clinical confusion, laboratories should: (1) clearly document which assay method was used in patient results, (2) apply method-specific cut-offs for clinical decision-making, and (3) when possible, maintain consistency in methodology for individual patients to allow meaningful longitudinal trend assessment. Future harmonization efforts, such as those promoted by the European Crohn’s and Colitis Organisation (ECCO), are essential to establish reference standards and cross-platform conversion factors.

Cost considerations, existing instrumentation, and required clinical turnaround times should guide final assay selection in routine practice. For most clinical settings, either method is acceptable when platform-specific thresholds are rigorously applied, ensuring reliable non-invasive monitoring of UC disease activity.

### 4.3. Future Directions

To improve the reliability and standardization of fecal calprotectin testing across clinical settings, several priorities for future research emerge from this work. First, larger, prospective multicenter studies enrolling diverse UC populations (including untreated patients, those on conventional therapy, and those receiving biologics) are needed to validate the method-specific cut-off values reported here and establish whether cut-offs differ by patient subgroup or treatment context. Such studies should employ standardized protocols for sample collection, storage, timing of endoscopic evaluation, and biomarker measurement.

Second, efforts to develop and validate cross-platform conversion equations or calibration factors would enable more seamless clinical implementation and allow meaningful comparison of historical data obtained using different assays. Standardization bodies including the International Organization for Standardization (ISO) and professional societies such as ECCO should prioritize harmonization of FC assay calibration, reporting units, and quality control standards across manufacturers.

Third, future investigations should explore whether incorporating method-specific FC measurements into machine learning or artificial intelligence models could enhance prediction of treatment response, risk of relapse, or need for treatment escalation—areas where FC has shown promise but remains imperfectly predictive. Integration of FC with other biomarkers (such as fecal lactoferrin, C-reactive protein, or fecal immunological biomarkers) may further refine disease activity assessment.

Fourth, mechanistic studies examining the physiological basis for the inter-method differences observed here (including differences in antibody specificity, sample extraction efficiency, and calibration approaches) would improve understanding and potentially guide assay optimization. Finally, real-world effectiveness studies examining the impact of implementing method-specific FC thresholds in clinical practice settings on clinical outcomes, treatment decisions, and patient management would provide evidence for best practices in IBD monitoring. Collectively, these efforts should enhance the clinical utility of fecal calprotectin as a cornerstone non-invasive biomarker in ulcerative colitis management.

## 5. Conclusions

Both Calprest ELISA and Liaison CLIA are effective for measuring fecal calprotectin and assessing disease activity in ulcerative colitis. Calprest offers broader analytical range and slightly better discrimination at higher FC levels, while Liaison provides faster, automated analysis with robust reproducibility. Despite minor inter-method variability, each test is suitable for clinical use when method-specific thresholds are applied. Ensuring consistency in test selection and interpretation is essential for optimizing non-invasive disease monitoring strategies in UC.

## Figures and Tables

**Table 1:** Patient Demographics and Clinical Characteristics.

Characteristic	
Age (years), median (IQR)	Median = 42, IQR = 19.5
Sex, n (%)	
Female	24 (60%)
Male	16 (40%)
Disease duration, years	Median = 5, IQR = 6.5
Disease extent, n (%)	
Proctitis	60.0% (E1)
Left-sided	17.14% (E2)
Extensive	22.86% (E3)
Current medications, n (%)	
5-ASA	37.5%
Biologics	100%
Mayo endoscopic scores, n (%)	
0 (remission)	7.89%
1 (mild)	26.32%
2 (moderate)	47.37%
3 (severe)	18.42%

**Table 2:** Analytical and Operational Characteristics of Calprest ELISA and Liaison CLIA Methods for Fecal Calprotectin Measurement.

Feature	Calprest ELISA	Liaison CLIA
Detection Method	Enzyme-linked immunosorbent assay (ELISA)	Chemiluminescent immunoassay (CLIA)
Measuring Range	30–1800 µg/g	20–800 µg/g
Assay Time	~1.5–2 h	~35 min
Automation	Manual/Semi-automated	Fully automated
Precision (CV%)	~4–7%	~3–6%
Throughput	Batch (96 samples)	Continuous loading
Sensitivity/Specificity	95%/93%	93%/96%
Sample Preparation	Manual homogenization	Integrated extraction

**Table 3:** Descriptive Statistics of Fecal Calprotectin Concentrations by Liaison and Calprest Methods.

Test	Mean (µg/g)	Median (µg/g)	SD (µg/g)	Min (µg/g)	Max (µg/g)	IQR (Q1–Q3, µg/g)
Liaison	288.24	149.00	300.84	5.00	800.00	516.20
Calprest	242.26	102.00	272.76	1.00	791.00	397.00

**Table 4:** Method-Specific Cut-off Values for FC Concentrations to Discriminate Remission and Inflammation.

Comparison	Cut-off (µg/g)	Method	Youden Index
Remission vs. Inflammation (Mayo 0 vs. ≥1)	47.95	Liaison	0.583
	65	Calprest	0.608
Improvement vs. Active Inflammation (Mayo ≤ 1 vs. ≥2)	69.55	Liaison	0.369
	125	Calprest	0.381

## Data Availability

The raw data supporting the conclusions of this article will be made available by the authors on request.

## Abbreviations

The following abbreviations are used in this manuscript:

AUC	Area Under the Curve
CLIA	Chemiluminescent Immunoassay
CV	Coefficient of Variation
ELISA	Enzyme-Linked Immunosorbent Assay
ECCO	European Crohn’s and Colitis Organisation
FC	Fecal Calprotectin
IBD	Inflammatory Bowel Disease
IBS	Irritable Bowel Syndrome
IQR	Interquartile Range
ROC	Receiver Operating Characteristic
SD	Standard Deviation
UC	Ulcerative Colitis

## References

[1] Zhang Y.Z., Li Y.Y. (2014). Inflammatory bowel disease: Pathogenesis. World J. Gastroenterol..

[2] Benítez J.M. (2015). Faecal calprotectin: Management in inflammatory bowel disease. World J. Gastrointest. Pathophysiol..

[3] Sipponen T., Kolho K.L. (2015). Fecal calprotectin in diagnosis and clinical assessment of inflammatory bowel disease. Scand. J. Gastroenterol..

[4] Mahler M., Bentow C., Serra J., Fritzler M.J. (2016). Detection of autoantibodies using chemiluminescence technologies. Immunopharmacol. Immunotoxicol..

[5] Khan M., Shah S.H., Salman M., Abdullah M., Hayat F., Akbar S. (2023). Enzyme-Linked Immunosorbent Assay versus Chemiluminescent Immunoassay: A General Overview. Glob. J. Med. Pharm. Biomed. Update..

[6] Macias-Muñoz L., Frade-Sosa B., Iniciarte-Mundo J., Hidalgo S., Morla R.M., Gallegos Y., Sanmarti R., Auge J.M. (2022). Analytical and clinical evaluation of DiaSorin Liaison^®^ Calprotectin fecal assay adapted for serum samples. J. Clin. Lab. Anal..

[7] Juricic G., Brencic T., Tesija-Kuna A., Njegovan M., Honovic L. (2019). Faecal calprotectin determination: Impact of preanalytical sample treatment and stool consistency on within- and between-method variability. Biochem. Med..

[8] Ikeya K., Hanai H., Sugimoto K., Osawa S., Kawasaki S., Iida T., Maruyama Y., Watanabe F. (2016). The Ulcerative Colitis Endoscopic Index of Severity More Accurately Reflects Clinical Outcomes and Long-term Prognosis than the Mayo Endoscopic Score. J. Crohns Colitis..

[9] Srinivas M., Eyre R., Ellis R., Viney S., Basumani P., Bardhan K. (2012). PTU-243 Faecal calprotectin (FC) assays: Comparison of four assays with clinical correlation. Gut.

[10] Fiorino G., Danese S., Peyrin-Biroulet L., Sans M., Bonelli F., Calleri M., Zierold C., Pollastro R., Moretti F., Malesci A. (2022). LIAISON^®^ Calprotectin for the prediction of relapse in quiescent ulcerative colitis: The EuReCa study. United Eur. Gastroenterol. J..

[11] Cannatelli R., Bazarova A., Zardo D., Nardone O.M., Shivaji U., Smith S.C.L., Gkoutos G., Ricci C., Gui X.S., Ghosh S. (2021). Fecal Calprotectin Thresholds to Predict Endoscopic Remission Using Advanced Optical Enhancement Techniques and Histological Remission in IBD Patients. Inflamm. Bowel Dis..

[12] Vicente-Steijn R., Jansen J.M., Bisheshar R., Haagen I.A. (2020). Analytical and clinical performance of the fully-automated LIAISONXL calprotectin immunoassay from DiaSorin in IBD patients. Pract. Lab. Med..

[13] Pelkmans L.P.J., de Groot M.J.M., Curvers J. (2019). Analytical Performance and Clinicopathologic Correlation of Four Fecal Calprotectin Methods. Am. J. Clin. Pathol..

[14] D’Amico F., Rubin D.T., Kotze P.G., Magro F., Siegmund B., Kobayashi T., Olivera P.A., Bossuyt P., Pouillon L., Louis E. (2021). International consensus on methodological issues in standardization of fecal calprotectin measurement in inflammatory bowel diseases. United Eur. Gastroenterol. J..

[15] Stevens T.W., Gecse K., Turner J.R., de Hertogh G., Rubin D.T., D’Haens G.R. (2021). Diagnostic Accuracy of Fecal Calprotectin Concentration in Evaluating Therapeutic Outcomes of Patients With Ulcerative Colitis. Clin. Gastroenterol. Hepatol..

